# Methyl-CpG binding domain proteins inhibit interspecies courtship and promote aggression in *Drosophila*

**DOI:** 10.1038/s41598-017-05844-6

**Published:** 2017-07-14

**Authors:** Tarun Gupta, Hannah R. Morgan, Jonathan C. Andrews, Edmond R. Brewer, Sarah J. Certel

**Affiliations:** 1 Neuroscience Graduate Program, The University of Montana, Missoula, MT United States; 2 Division of Biological Sciences, The University of Montana, Missoula, MT United States

## Abstract

Reproductive isolation and speciation are driven by the convergence of environmental and genetic variation. The integration of these variation sources is thought to occur through epigenetic marks including DNA methylation. Proteins containing a methyl-CpG-binding domain (MBD) bind methylated DNA and interpret epigenetic marks, providing a dynamic yet evolutionarily adapted cellular output. Here, we report the *Drosophila* MBD-containing proteins, dMBD-R2 and dMBD2/3, contribute to reproductive isolation and survival behavioral strategies. *Drosophila melanogaster* males with a reduction in dMBD-R2 specifically in octopamine (OA) neurons exhibit courtship toward divergent interspecies *D. virilis* and *D. yakuba* females and a decrease in conspecific mating success. Conspecific male-male courtship is increased between dMBD-R2-deficient males while aggression is reduced. These changes in adaptive behavior are separable as males with a hypermethylated OA neuronal genome exhibited a decrease in aggression without altering male-male courtship. These results suggest *Drosophila* MBD-containing proteins are required within the OA neural circuitry to inhibit interspecies and conspecific male-male courtship and indicate that the genetically hard-wired neural mechanisms enforcing behavioral reproductive isolation include the interpretation of the epigenome.

## Introduction

A long-standing challenge in evolutionary biology is to understand the molecular basis of adaptive, divergent phenotypes. Between recently diverged species, mating behavior can either impede or promote reproductive isolation. Like reproductive behaviors, the social behavior aggression also relies on sexual and species discrimination^1–4^. In males, the choice between courtship of conspecific females and aggression towards conspecific males requires rapid decision-making based on the integration of external information and internal states^5–7^. While the internal state of an organism requires reliable gene transcription from the stable genome of each cell, navigating a dynamic environment can involve flexibility in gene expression. Epigenetic mechanisms that alter gene activity including chromatin remodeling and DNA methylation are responsive to external pressures^8–11^. Here we test the hypothesis that epigenetic mechanisms influence sex and species discrimination by examining the function of methyl-CpG-binding domain (MBD) proteins as a substrate for selection within a key subset of neuromodulatory neurons.

Key neuromodulators include biogenic amines such as serotonin, dopamine, and octopamine (OA, the invertebrate structural analogue of norepinephrine) that promote or regulate aggression and reproductive behaviors in numerous systems ranging from crustaceans to primates^12–15^. We previously demonstrated that OA plays a critical role in regulating male aggression and the choice between aggression and courtship by directly modulating male pheromonal stimuli received by the gustatory receptor 32a (Gr32a)^16^. Eliminating Gr32a function itself also reduces conspecific male aggression, increases conspecific male-male courtship and is required to inhibit interspecies courtship^16–19^. These results demonstrated that direct modulation of sex- and species-specific sensory information by OA is a critical component of male adaptive behavior^16^.

With this foundation, we asked if *Drosophila* MBD proteins interpret critical genome-mediated biological processes within OA neurons that facilitate adaptive behavior. MBD proteins were initially classified as epigenetic regulators due to their ability to interact with histone deacetylase (HDAC)-containing complexes and bind 5-methylcytosine (5mC)^20^. Cytosine methylation was recently confirmed in *Drosophila* ^21–24^ and the *Drosophila* genome encodes at least two MBD-containing proteins, dMBD-R2 and dMBD2/3^25, 26^. The dMBD2/3Δ splice variant preferentially recognizes 5mC-containing DNA through its MBD domain^26^, associates with components of the nucleosome remodeling and deacetylase (NuRD) complex^27^, and is present in adult tissues^27, 28^. While it has not been determined if dMBD-R2 binds 5mC, dMBD-R2 also is expressed in the adult, and is part of the multi-subunit chromatin remodeling NSL (non-specific lethal) complex, which regulates gene expression at genome wide levels^26^. As a group, MBD proteins play a major role in determining the transcriptional state of the genome by coordinating crosstalk between DNA methylation, histone modifications and chromatin organization. These functions have been commonly exploited in human disease and behavior. Here we ask, are genome-wide readout mechanisms a target in the evolution of divergent sex-specific behaviors?

By using behavioral assays and an RNAi based reduction of dMBD-R2 and dMBD-2/3 specifically in OA neurons, we demonstrate *Drosophila* MBD-containing proteins are required for evolutionarily driven adaptive behavior. Reducing the expression of MBD proteins in OA neurons decreased male aggression toward a conspecific male, increased conspecific male-male courtship, and elevated interspecies courtship levels. Our data suggests there are fitness costs related to the inability to inhibit interspecies courtship as successful conspecific mating was reduced in MBD-R2-and MBD-2/3-deficient males. As the observed MBD-induced behavioral changes could occur as a result of methylation-dependent or methylation-independent interactions, we examined mate discrimination and male behavioral choice following hypermethylation of the OA neuron genome. Males with a hypermethylated genome exhibited a decrease in aggression without altering male-male courtship. Together, these results indicate dMBD-R2 function is required in male OA neurons to maintain or inhibit a transcriptional program that enables the correct response to species-specific pheromonal cues. We proposed that epigenetic mechanisms interpreted by MBD proteins are required for male social behavior and pave the way to address how the selective utilization of the OA neuronal genome and potential shifts in gene expression in response to sensory stimuli, are coordinated at the epigenome level.

## Materials and Methods

### Drosophila Husbandry and Stocks

All flies were reared on standard cornmeal-based fly food. Unless noted otherwise, during developmental and post-eclosion, flies were raised at 25 °C, ~50% humidity and a 12:12 hr light-dark cycle (1400 ± 200 lx white fluorescent light) in humidity and temperature controlled incubators.

The following stocks were used: Canton-S (BL 64349), *tub-Gal80* ^*ts*^ (*BL 7019*), *UAS-CD8:GFP* (BL 5130), *20XUAS-IVS-mCD8:GFP* (BL 32194), *UAS-dMBD-R2-RNAi* (BL 30481), and *UAS-dMBD2/3-RNAi* (BL 35347) were obtained from the Bloomington Stock Center (Bloomington, IN). *Drosophila virilis* (15010–1051.00) and *D. yakuba* (14021.0261.38) was received from the Drosophila Species Stock Center (San Diego, CA). *Cha-Gal80* was a gift from T. Kitamoto and Jay Hirsh generously provided the *Tdc2-Gal4* line.

### Aggression Assays

For aggression and inter-male courtship analysis, male pupae were isolated and aged individually in 16 × 100 mm borosilicate glass tubes containing 1.5 ml of standard food medium described above. A dab of white or blue acrylic paint was applied to the thorax of two-day old males under CO_2_ anesthesia for identification purposes. Flies were returned to their respective isolation tubes for a period of at least 24 hours to allow recovery from handling and anesthesia. For aggression testing, pairs of 3–5 day old, socially naïve adult males were placed in 12-well polystyrene plates (VWR #82050-930) as described previously^29^. The number of lunges and wing threats were counted for 30-mins after the first lunge. For temperature sensitive *tub-Gal80* ^*ts*^ experiments, flies were raised at 18–19 °C through embryonic, larval and pupal stages. Individual pupae were allowed to eclose in isolation. 2–3 day old adult males in isolation vials were transferred to 30 °C for 24–36 hrs for Gal80^ts^ inactivation. 30-min prior to behavioral testing, flies were moved to 25 °C for recovery. Aggression and inter-male courtship were assayed at 25 °C and ~45–50% humidity levels in standard polystyrene chambers as described earlier.

### Scoring

All aggression was assayed within first two hours of lights ON time (Zeitgeber hours 0–2) and scored manually using iMovie ‘09, version 8.0.6. Total number of lunges and wing threat behaviors were scored for a period of 30 minutes after the first lunge according to the criteria established previously^30, 31^. The delay between the assay start time and the first lunge was used for calculating the delay to aggression onset (or latency to lunge). Dominance was established after 3 consecutive lunges followed by retreat of the second male off of the food cup. Male-male courtship was quantified by measuring the number of unilateral wing extensions (singing) within the aggression paradigm. Single wing extensions were counted prior to the first lunge as well as after the onset of aggression for a period of 30 minutes. All graphs were generated with Graphpad Prism and Adobe Illustrator CS5.

### Interspecies Courtship

For the interspecies courtship preference assay, a single 3–5 day old socially naïve control (Canton S), transgenic controls, or a *tdc2-Gal4;UAS-dMBD-R2-RNAi* male was paired with one 5–7 day old socially naïve conspecific female *(D. melanogaster)*, and one 7–8 day old socially naïve *D. virilis* female or one 3 day old socially naïve *D. yakuba* female. Courtship was quantified as the number of single wing extensions and abdominal bends. Additional courtship parameters were recorded including; latency to courtship or first unilateral wing extension, duration of each wing extension, total time spent courting each female, and the number of copulatory abdominal bends. Courtship index (C.I.) is defined as the total time a male courted either female divided by the total scoring period, in the event a successful mating event does not occur^32, 33^. Reproductive behaviors were scored for a total period of 10 minutes (600 seconds) or up to the point of successful mating event, whichever came earlier.

### Statistics

One-way analysis of variance (ANOVA) with Tukey’s multiple-comparison test was performed in cases of three or more comparison groups, and a standard pairwise t-test in case of only two comparisons. If data did not meet parametric assumptions, Kruskal-Wallis Test with Dunn’s multiple comparison was used unless otherwise specified. To identify outliers, the Rout method was used with Q set to 0.2%^34^.

### Negative Geotaxis

Newly eclosed males were aged 2–3 days at ~25 C, 45% humidity. Subsequently, males in groups of ~20 were carefully transferred via aspirator to a centimeter-marked polystyrene vial as previously described^35^. After a 15-min recovery period, the flies were knocked to the bottom of the vial by three taps. The subsequent climbing behavior was continually recorded and the three taps delivered a total of six times with a minute recovery time between taps. After transferring the video to a computer, the distance climbed by each fly per trial was averaged resulting in each trial equaling one data point. Significance was determined using the Kruskal-Wallis test with Dunn’s multiple comparison.

### Immunohistochemistry

Adult male dissected brains were fixed in 4% paraformaldehyde (Electron Microscopy Sciences) for 25 minutes and labeled using a modification of protocols previously described^36^. The following primary antibodies were used: anti-bruchpilot (mAb nc82, 1:30, Developmental Studies Hybridoma Bank)^37^, monoclonal rabbit anti-GFP (1:200, Molecular Probes), and rabbit anti-Tdc2 (1;100, Covalab). Secondary antibodies include Alexa Fluor 488-conjugated goat anti-rabbit and Alexa Fluor 647-conjugated donkey anti-mouse (Molecular Probes). Images were collected on an Olympus Fluoview FV1000 laser scanning confocal mounted on an inverted IX81 microscope and processed using ImageJ (NIH) and Adobe Photoshop (Adobe, CA). Brains in Supplemental Fig. 4 were labeled with diluted primary and secondary antibodies from the same aliquot, imaged with the same settings, and processed without modifications with ImageJ. Cell fluorescence was measured as previously described^38^.

## Results

### MBDR2-deficient males exhibit high-levels of interspecies courtship

To test the hypothesis that methyl-binding domain proteins contribute to behavioral reproductive isolation and species divergence, we examined the courtship behavior of *D. melanogaster* males with reduced MBD protein levels directed toward two drosophilids, *D. virilis* and *D. melanogaster*. As *D. virilis* and *D. melanogaster* diverged ∼40 million years ago (mya) and prior reports suggested wild-type *D. melanogaster* males do not court *D. virilis* females^17^, we asked if MBD proteins were under adaptive selection for their role in species-specific behaviors and if *D. melanogaster* males with decreased dMBD-R2 levels would display deficits in these behaviors.

**Figure 1:**
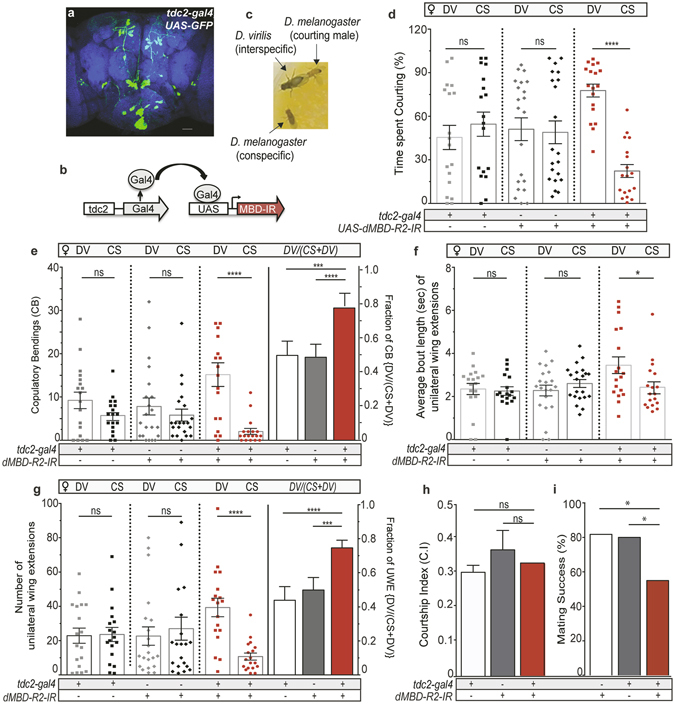
dMBD-R2-deficient males exhibit high-levels of interspecies courtship. (**a**) OA neurons identified by GFP expression (green) in a wildtype adult *tdc2-Gal4/20X-UAS-6XGFP* brain (neuropil labeled by nc82 (anti-bruchpilot) immunofluorescence, blue). (**b′**) dMBD-R2 targeted knockdown in OA neurons through the *UAS-Gal4* system. (**c**) Courtship preference assay: one *D.melanogaster* male courting a *D. virilis* female in the presence of a conspecific female. (**d–g**) Quantification of courtship behavioral patterns displayed by males with a reduction in dMBD-R2 (*tdc2-Gal4*;*UAS-dMBD-R2-RNAi*) and transgenic control males towards conspecific (CS) and interspecific *(D. virilis*; labeled DV) females. Mann-Whitney test was used unless otherwise specified, ****p < 0.0001, ***p < 0.001, **p < 0.01, *p < 0.05. n = 26 (*UAS-MBD-R2-IR*/+), 18 (*Tdc2-Gal4/*+), and 18 (*tdc2-Gal4*;*UAS-MBD-R2-IR*). (**d**) Males with reduced levels of dMBD-R2 in OA neurons spent the majority of the time (77.7%) courting DV females as compared to transgenic controls. (**e**) The average number and the proportion of abdomen bends toward DV females are significantly higher in dMBD-R2 deficient males than copulation attempts to conspecific females. **(f)** The duration of wing extensions to DV and CS females did not differ in transgenic control males (^ns^ *P* = 0.8512; Kruskal-Wallis Test with Dunn’s multiple comparison). The average bout length of unilateral wing extensions towards DV females as compared to CS females increased in dMBD-R2 deficient males (*P = 0.0364). (**g**) The number of unilateral wing extensions (singing) toward interspecific females was higher than towards conspecific females in dMBD-R2-reduced males as compared to control males. **(h)** Courtship index (C.I.) calculated as total time spent courting either female as a fraction of total scoring period did not differ between experimental and control males (^*ns*^ *P* = *0.6883*). (**i**) The percent of assays that resulted in a successful conspecific mating event was decreased significantly in *tdc2-Gal4*;*UAS-MBD-R2-IR* males as compared to transgenic controls (N-1 Chi-square test for proportions). n = 47 (*UAS-MBD-R2-IR/*+), 33 (*Tdc2-Gal4/*+), and 27 (*tdc2-Gal4*;*UAS-MBD-R2-IR*). Error bars indicate SEM.

Given the established role of octopamine in male social behavior and the subpar locomotive activity of males with manipulated MBD proteins pan-neuronally^39^, we asked if selectively reducing MBD levels in OA neurons altered the courtship behavior of *D. melanogaster* males towards interspecies females. Through the use of the Gal4-UAS system, *dMBD-R2* and *dMBD-2/3* levels were reduced by separately expressing UAS-driven inverted repeat transgenes (*UAS-dMBD-R2-RNAi* and *UAS-dMBD-2/3-RNAi)* under control of the *tdc2 (tyrosine decarboxylase (tdc2)-gal4* driver (Fig. 1a,b). In invertebrates, OA is synthesized from the amino acid tyrosine via the action of tyrosine decarboxylase (TDC) and tyramine β-hydroxylase (Tβh)^40^. The *Tdc2* gene encodes the neuronal TDC and the *tdc2-gal4* driver can be used to manipulate OA/TA neurons^41^. We previously demonstrated RNAi interference expression significantly decreases *dMBD-R2* and *dMBD-2/3* transcript levels in adult neurons using the pan-neuronal driver, *n-syb-Gal4* (36.79% and 26.84% respectively and no obvious defects in OA neuron structure or development occurs due to the reduction of MBD protein levels^39, 42^.

Therefore, we examined the courtship choice behavior of single socially naïve *D. melanogaster* males when placed with both a conspecific and *D. virilis* female as potential mating partners (Fig. 1c). This type of interspecies courtship paradigm provides the control or experimental male a choice between a conspecific or *D. virilis* female. Interactions between three flies at one time are minimal with the male courting one female or the other and the assay may provide an ethologically relevant situation as *D. virilis* and *D. melanogaster* have been reported to share urban habitats^43^. To measure reproductive behavior, we quantified the number and duration of unilateral wing extensions (singing), copulation attempts, and the percentage of time the male spent performing either of these courtship behaviors. In contrast to previous reports^17^, we found that socially naïve *D. melanogaster* wildtype (Canton S) males did court *D. virilis* females, however, wildtype males spent the majority of the assay period courting the conspecific female (Fig. S1a–e).

We next asked if dMBD-R2 deficient males (*tdc2-gal4;UAS-dMBD-R2-RNAi*, hereafter referred to as *tdc2*;*dMBD-R2-RNAi*) and transgenic controls preferentially court the conspecific or *D. virilis* female. Transgenic control (*tdc2-gal4/*+ and *UAS-dMBD-R2-RNAi/*+) males displayed courtship behaviors including unilateral wing extensions (the courtship song) and abdominal copulatory bending attempts to *D. virilis* females at levels reaching ~50% of time spent courting (Fig. 1d–g). To determine if courtship displays to the *D. virilis* female is exhibited by other transgenic controls, the behavior of *tryptophan hydroxylase(Trh)-Gal4* males that had previously been cantonized for six generations was quantified^44^ (Fig. S1f–k). *Trh-Gal4/*+ transgenic control males also displayed unilateral wing extensions and copulatory bends to both *D. virilis* and conspecific females suggesting increased interspecies courtship may be a characteristic of transgenic controls (Fig. S1g,i). In contrast, *tdc2;dMBD-R2-RNAi* males directed courtship behavior to *D. virilis* females ~80% of the total courtship time (Fig. 1d). Transgenic control males attempted copulation (abdomen bending) to both conspecific or *D. virilis* females, whereas males with reduced dMBD-R2 levels in OA neurons directed copulation attempts toward the *D. virilis* female at significantly higher levels than conspecific females (Fig. 1e).

By quantifying conspecific and interspecific single wing extensions as individual behaviors, we determined that, in the same manner as the abdominal bending behavior, males with reduced dMBD-R2 levels displayed a significant increase in the average number of wing extensions toward the *D. virilis* female as compared to transgenic controls (Fig. 1g). To examine if there was a quantitative difference in interspecies courtship behavior by experimental males, we measured courtship song duration. Although there was no difference in song duration directed toward either female by transgenic controls, the duration of the courtship song by *tdc2;dMBD-R2-RNAi* males was significantly longer to *D. virilis* females than conspecific females (Fig. 1f). Does this increase in time spent courting a non-receptive female impact the reproductive success of dMBD-R2 deficient males? Experimental males maintained the same level of courtship vigor as measured by the courtship index (C.I.) as transgenic controls (Fig. 1h), however we found a significant decrease in copulation rates between dMBD-R2 deficient males and the conspecific female as compared to transgenic controls (Fig. 1i).

Males deficient in a second *Drosophila* MBD-containing protein, dMBD-2/3 (*tdc2-Gal4;UAS-dMBD-2/3-RNAi*, Fig. S2a) hereafter referred to as *tdc2;dMBD-R2-RNAi*) also spent significantly more time courting the *D. virilis* female than the conspecific (Fig. S2b) with increased numbers of wing extensions toward the *D. virilis* female as compared to transgenic controls (Fig. S2d). Attempted copulations by *tdc2;dMBD-2/3-RNAi* males did not favor either female (Fig. S2c) and the courtship index was reduced (Fig. S2e). Consistent with the results above, the presence of an interspecies female significantly decreased the copulation rate of dMBD-2/3 deficient males to the conspecific female indicating there is a reproductive cost to the reduction of MBD-2/3 in OA neurons (Fig. S2f). Taken together, these results indicate males require the function of dMBD-containing proteins in OA neurons to possibly establish or maintain a transcriptional program that enables the correct response to species-specific pheromonal cues.

**Figure 2:**
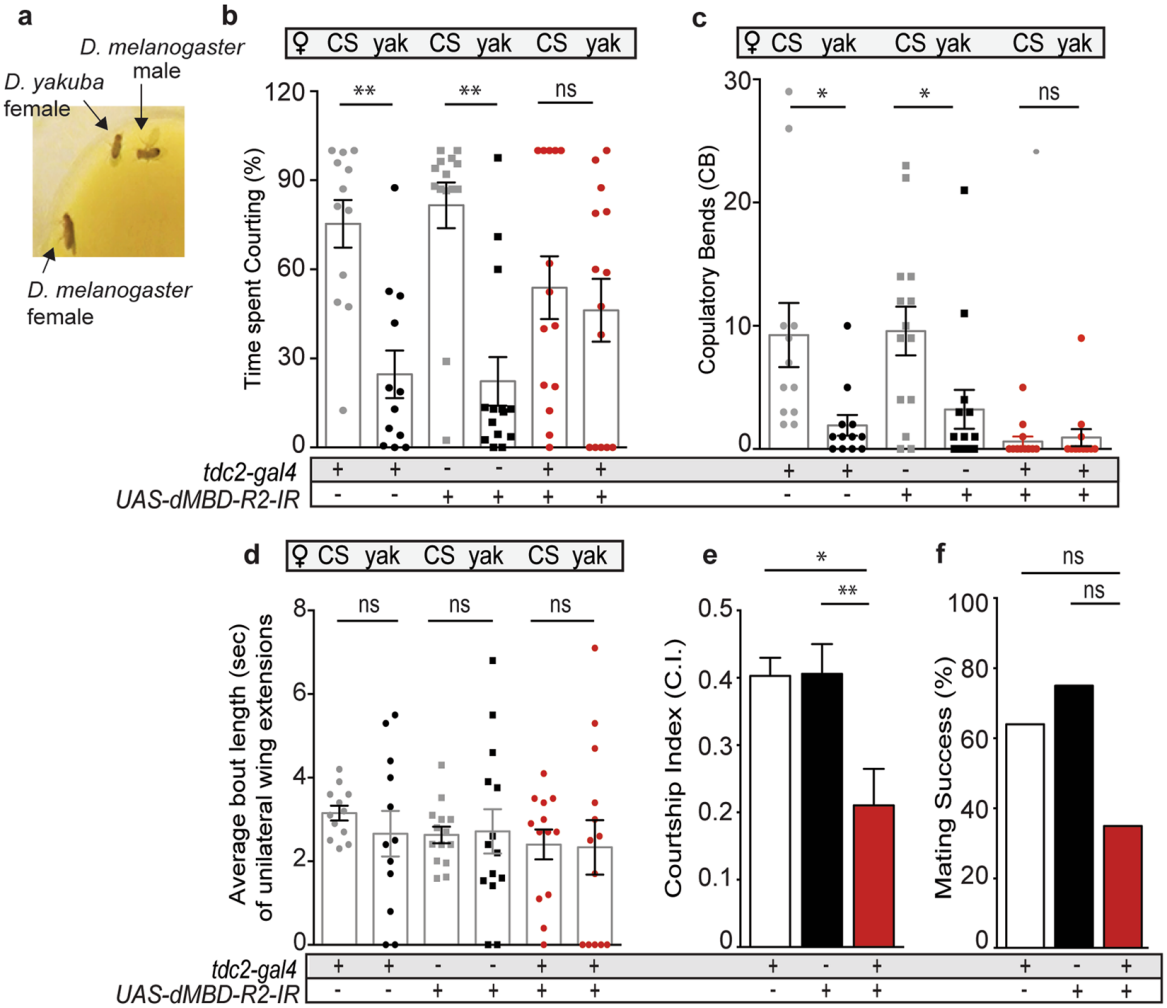
Males with reduced MBD-R2 levels in OA neurons exhibit high-levels of interspecies courtship toward *D*. *yakuba* females. (**a**) Courtship preference assay: one *D. melanogaster* male courting a *D. yakuba* female in the presence of a conspecific female. (**b–f**) Quantification of courtship behavioral patterns displayed by males with a reduction in dMBD-R2 (*tdc2-Gal4*;*UAS-dMBD-R2-RNAi*) and transgenic control males towards conspecific (CS) and interspecific (*D. yakuba*; labeled “yak”) females. One-way ANOVA with Tukey’s multiple comparisons test was used unless otherwise specified, ****p < 0.0001, ***p < 0.001, **p < 0.01, *p < 0.05. n = 14 (*UAS-MBD-R2-IR/*+), n = 12 (*Tdc2-Gal4/*+), and n = 14 (*tdc2-Gal4*;*UAS-MBD-R2-IR*). (**b**) Males with reduced levels of dMBD-R2 in OA neurons courted both females. Transgenic controls spent more time courting conspecific females than *D. yakuba*. (^ns^ *P* = 7.607). (**c**) The average number of abdomen bends toward *D*. *yakuba* and conspecific females do not differ in dMBD-R2 deficient males (^ns^ *P* = −0.3077). (**d**) The duration of wing extensions to *D. yakuba* and CS females did not differ in control and dMBD-R2-deficient males. (**e**) Courtship index (C.I.) was significantly lower in *tdc2-Gal4*;*UAS-dMBD-R2-RNAi* males versus controls. (**i**) The percent of assays that resulted in a successful conspecific mating event by *tdc2-Gal4*;*UAS-MBD-R2-IR* males was 35% which did not significantly differ from transgenic controls (N-1 Chi-square test for proportions). Error bars indicate SEM.

Previous reports suggested the large size of *D. virilis* females compared to conspecific females may attract males to attempt copulation more frequently^17^. To address this possibility, we placed a *D. yakuba* female with one conspecific female and a single experimental or control male (Fig. 2a). The *D. melanogaster* lineage split from *D. yakuba* approximately 5.1 +/− 0.8 million years ago (mya)^45^ and *D. yakuba* females are of comparable size to *D. melanogaster* females. Given a choice between a *D. yakuba* female and a conspecific, the same transgenic controls now preferentially court the conspecific (Fig. 2b–d). However, experimental males with reduced dMBD-R2 levels did not exhibit the same preference for the conspecific and instead directed courtship behavior towards both females. Neither the average number of wing extensions nor copulation attempts by *tdc2;dMBD-R2-RNAi* males were significantly different between the conspecific females and *D. yakuba* females (Fig. 2b,c).

Simply exchanging the *D. virilis* female for a *D. yakuba* surprisingly elicited a reduced level of courtship vigor (C.I.) by males deficient in MBD-R2 in OA (Fig. 2e) and mating success was reduced to 35% (Fig. 2f) suggesting there is a reproductive cost to the reduction of MBD-2/3 in OA neurons. Taken together, these results indicate dMBD-R2 function is required in male OA neurons to potentially establish or maintain a transcriptional program that enables the correct response to species-specific pheromonal cues.

### Reducing *Drosophila* MBD protein levels decreases male aggression and increases male-male courtship

Recent studies indicate that alterations in the levels of the MBD family member, methyl CpG binding protein 2 (MeCP2), can impact aggression and defense behaviors. In mice, aggression is increased when MeCP2 function is removed from serotonergic neurons and separately in a subset of hypothalamic neurons^46, 47^. However, a mild transgenic overexpression of MeCP2 can increase or decrease depending on the murine genetic background^48^. Likewise in humans, specific MeCP2 SNPs and alterations in MeCP2 levels have been associated with impulse control deficits and abnormal aggression in schizophrenia cohorts as well as in patients with Rett syndrome and MeCP2-duplication syndrome^48–50^. Therefore, we examined the aggressive behavior of *D. melanogaster* males to determine if the function of MBD proteins in survival behaviors is evolutionarily conserved.

**Figure 3:**
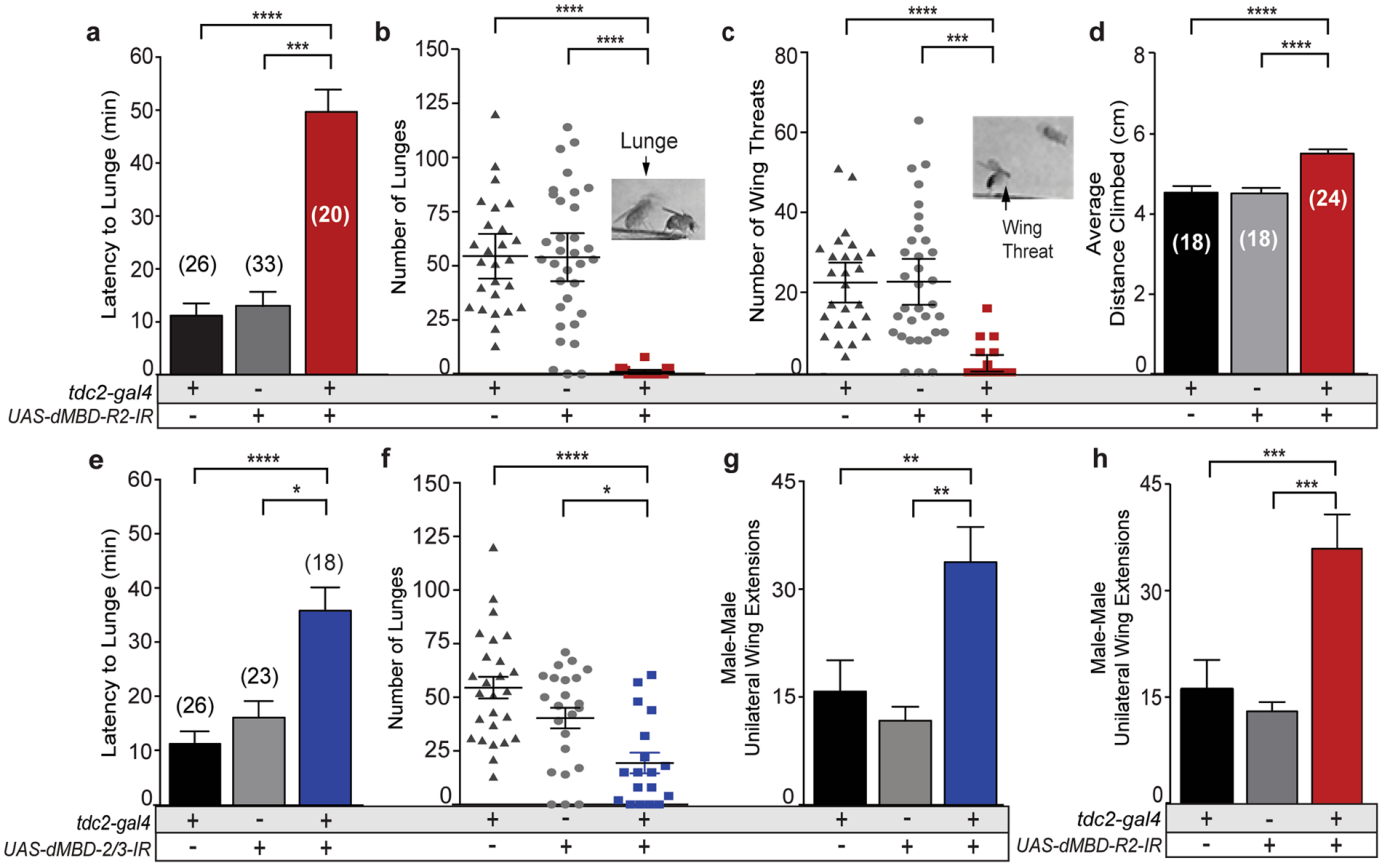
Reducing MBD proteins in OA neurons decreases male aggression and increases male-male courtship. (**a–d**) Fights between males with reduced dMBD-R2 levels in OA neurons *(tdc2-Gal4; UAS-MBD-R2-IR)* and individual transgenic controls, *UAS-dMBD-R2-RNAi/*+ or *tdc2-Gal4/*+. **(a)** Latency to first lunge was significantly increased in *tdc2-Gal4;UAS-dMBD-R2-RNAi* males as compared to controls (****p < 0.0001, ***p < 0.001, **p < 0.001, *p < 0.05; Kruskal Wallis with Dunn’s multiple comparison test except where noted). The n value for each genotype is 26, 33, and 20. (**b**) Males with reduced dMBD-R2 levels in OA neurons lunged less as compared to controls; inset illustrates a male lunging (arrow). (**c**) dMBD-R2-deficient males display reduced wing threats as compared to transgenic controls; inset illustrates an aggressive bilateral wing extension (arrow). (**d**) The number of courtship patterns measured as unilateral wing extensions was significantly higher in pairs of dMBD-R2-deficient males. (**e–g**) Fights between males with reduced dMBD-2/3 levels in OA neurons *(tdc2-Gal4;UAS-dMBD-2/3-RNAi)* and individual transgenic controls, *UAS-dMBD-2/3-RNAi/*+ or *tdc2-Gal4/*+. (**e**) The latency to lunge was increased in MBD-2/3 deficient males (*tdc2-Gal4/+*;*UAS-dMBD-2/3-RNAi/*+) as compared to controls. The n value for each genotype is 26, 23, and 18. (**f**) Males with reduced dMBD-2/3 levels performed less lunges as compared. (**g**) *Tdc2-Gal4;UAS-dMBD-2/3-RNAi* males exhibited an increase in male-male courtship as measured by unilateral wing extensions. Error bars indicate SEM.

Pairs of *tdc2*;*dMBD-R2-RNAi* males, *tdc2-Gal4*;*UAS-dMBD-2/3-RNAi* males, and transgenic controls were placed in an aggression chamber^16, 51^ and multiple parameters were quantified including; latency to the first lunge, total numbers of lunges, and total number of agonistic wing threats. Males with decreased dMBD-R2 levels exhibited a significant reduction in aggressive behavior as demonstrated by decreases in lunge number and wing threats and an increase in the latency to initiate aggression as compared to controls (Fig. 3a–c). We previously determined the waking activity levels of *tdc2;dMBD-R2-RNAi* males does not differ from controls^39^. Here we demonstrate the decrease in male aggression does not reflect deficits in geotaxis behavior (Fig. 3d) as experimental males are more active in the negative geotaxis assay. A reduction in lunge number and an increase in latency were also observed in males with reduced dMBD-2/3 levels (Fig. 3e,f).

A second explanation for a decrease in aggression may be that males are engaging in an alternative behavior. Within the allotted fight assay time, interactions between control male pairings include low levels of male-male courtship as well as aggression patterns. We quantified male-male courtship by measuring unilateral wing extensions (the courtship song) and determined that males with reduced dMBD-2/3 or dMBD-R2 levels in OA neurons (*tdc2;dMBD-R2-RNAi, tdc2;dMBD-2/3-RNAi*) displayed a significant increase in male-male courtship (Fig. 2g,h), a phenotype also observed in males that lack OA^5, 16, 36^. These results suggest *Drosophila* MBD-containing proteins are required to promote conspecific aggression while inhibiting conspecific male-male courtship. Using a second *UAS-dMBD-R2-RNAi* line (dMBD-R2-RNAi^27029^), we confirmed wildtype levels of aggression require MBD-R2 function (Fig. S3a–c). While male-male courtship does not increase in *tdc2;dMBD-R2-RNAi* ^*27029*^ deficient males, (Fig. S3d), this result may reflect differences in the strength of the two dMBD-R2-RNAi lines. Due to the considerable decrease in aggression, we subsequently focused our attention on dMBD-R2.

### Behavioral-specific requirements for the epigenetic regulation of OA neuron function

**Figure 4:**
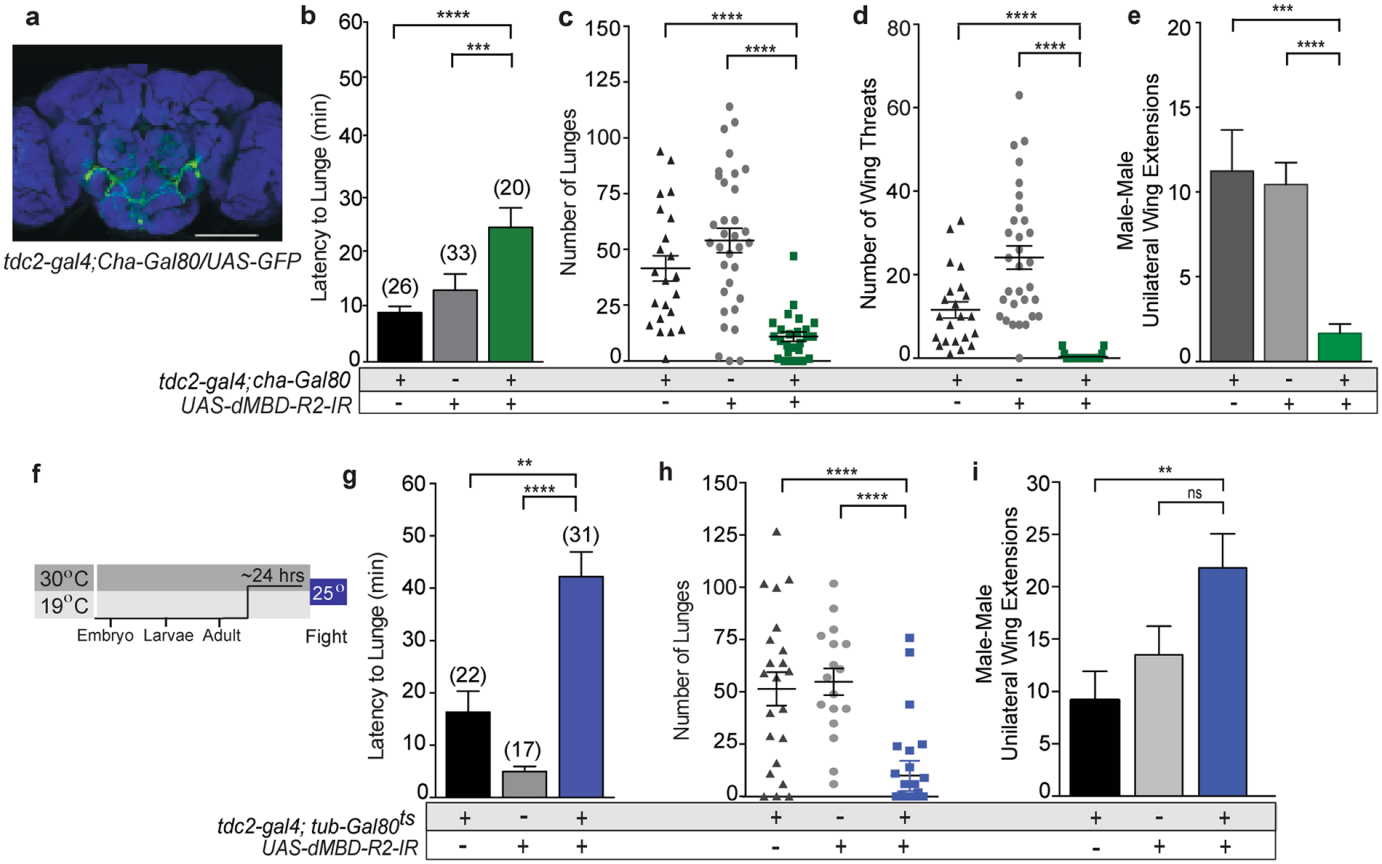
dMBD-R2 is required in the adult to promote male aggression. (**a**) A subset of OA neurons express GFP in the *tdc2-gal4/UAS-mCD8:GFP;Cha-Gal80* male. (**b**–**d**) Fights between males with reduced dMBD-R2 levels in a subset of OA neurons *(tdc2-Gal4/*+; *UAS-dMBD-R2-RNAi/Cha-Gal80)* and individual transgenic controls, *UAS-dMBD-R2-RNAi/*+ and *tdc2-Gal4/*+;*Cha-Gal80/*+. **(b)** The latency to lunge was significantly increased in experimental males as compared to controls (****p < 0.0001, ***p < 0.001, **p < 0.01; all statistical tests are Kruskal Wallis with Dunn’s multiple comparison test except where noted. (**c**) dMBD-R2-deficient males (*tdc2-Gal4/*+;*UAS-dMBD-R2-RNAi/Cha-Gal80*) exhibited a significant reduction in the number of lunges as compared to control males. (**d**) Experimental males display a significant reduction in the number of wing threats. (**e**) The number of male-male wing extensions was significantly lower in pairs of *tdc2-Gal4/*+; *UAS-dMBD-R2-RNAi/Cha-Gal80* males as compared to transgenic control pairs. (**f**) *tdc2-Gal4;UAS-dMBD-R2-RNAi/tub-Gal80* ^*ts*^ males were reared at 19 °C to prevent a reduction in dMBD-R2 until shifted to 30 °C prior to 24–36 hrs fighting in the aggression assay at 25 °C. **(g**–**i)** Fights between males with an adult-specific reduction of dMBD-R2 levels in OA neurons *(tdc2-Gal4*;*UAS-dMBD-R2-RNAi/tub-Gal80* ^*ts*^) and transgenic controls, *UAS-MBD R2-IR/*+ and *tdc2-Gal4;tub-Gal80* ^*ts*^. (**g**) The latency to lunge was significantly increased in males with adult-specific dMBD-R2-knockdown as compared to controls. (**h**) dMBD-R2 adult deficient experimental males exhibited a significant reduction in the number of lunges as compared to controls. (**i**) Male-male courtship events were not significantly different between the *UAS-MBD-R2-IR* control and dMBD-R2 adult deficient males. Error bars indicate SEM.

Aggression and courtship are for the most part mutually exclusive behaviors^51–54^, yet both are altered by a dMBD-R2 reduction in the entire OA neuronal population. To ask if the requirement for dMBD-R2 can be refined to a smaller OA neuron population and if reproductive and aggressive behaviors are separable, we used the Gal4 repressor GAL80 to selectively prevent dMBD-R2 reduction in the subset of OA neurons. Adding the *choline acetyltransferase* (*cha*)-Gal80 transgene (*tdc2-gal4;cha-Gal80/UAS-6XGFP)* limits the number of OA neurons with Gal4 activity to neurons within the suboesophageal zone (SEZ) and the ventrolateral cluster (OA-VL1 and OA-VL2) (Fig. 4a). Previous studies indicate OA is required in a subset of these neurons for male aggression^55^. Therefore, we predicted that males with reduced *dMBD-R2* levels in this OA neuronal subset would exhibit a decrease in aggression only.

All quantified aggression parameters including latency to lunge, lunge number, and the number of wing threats by experimental males (*tdc2;cha-Gal80/dMBD-R2-RNAi)* were significantly reduced as compared to controls (Fig. 4b–d). However, experimental males exhibited very low levels of male-male courtship (Fig. 4e). These results suggest the male-male courtship exhibited by *tdc2;dMBD-R2-RNAi* males in Fig. 1 is not a compensatory behavior that occurs due to reduced aggression, but rather may occur as a result of epigenetic alterations within OA neurons that are part of a courtship network. These observations are consistent with previous reports indicating distinct subsets of OA neurons and separable Gr32a-expressing populations regulate male aggression and courtship^5, 16^ and provide specific neuronal populations to analyze gene expression differences. To analyze if the production of OA itself may be a target of MBD proteins, we quantified the expression of Tdc2 in specific OA neurons. Based on quantification of immunofluorescence we found differences on a neuron-by-neuron basis (Fig. S4a–c) suggesting the possibility of different target genes among unique OA neuronal subsets.

### dMBD-R2 is required in adult OA neurons for male social behavior

Previous studies have determined MBD proteins play a role in interpreting the neuronal chromatin state during development, signaling, and stress responses^56–59^. In *Drosophila*, dynamic changes in chromatin structure initiate a series of transcriptional cascades during larval-pupal and pupal-adult metamorphosis^60–63^. Therefore, the observed deficits in male social behavior may reflect changes in OA neuronal differentiation that occur prior to the adult assay. To temporally restrict expression of the dMBD-R2-RNAi transgene and thereby ask if the function of dMBD-R2 is required in adult neurons, we used the temperature-sensitive mutant Gal80^ts^. Gal80^ts^ represses Gal4 activity at 19 °C but is not active at 30 °C^64^. In the following experiments, *tdc2*;*tub-Gal80* ^*ts*^ */dMBD-R2-RNAi* progeny were raised at 19 °C to prevent *dMBD-R2-RNAi* expression and subsequently, shifted to 30 °C for 24–36 hours prior to transference into the fight chamber (Fig. 4f).

When dMBD-R2 levels were reduced only during adulthood, the aggression of *tdc2*; *tub-Gal80* ^*ts*^ */dMBD-R2-RNAi* males were also reduced. Experimental males displayed a significant reduction in lunge number and a delay in the onset of aggression (Fig. 4g,h). Although dMBD-R2 adult deficient males exhibited a decrease in lunges, the experimental males did interact with each other with male-male courtship frequently an outcome of the interactions (Fig. 4i). These results indicate a role for dMBD-R2 in interpreting the genomic landscape of OA neurons in the adult and provide the ability to analyze gene-environment interactions within a refined temporal framework.

### Hypermethylation of the OA neuron genome decreases aggression without altering courtship

Although the distribution of 5 mC methylation in the *Drosophila* genome can be characterized as sparse, one of the conserved functions of MBD proteins is to bind methylated DNA. One approach to examine whether dMBD-R2 exerts its effects on aggression through methylation-dependent interactions is to hyper-methylate the OA neuron genome and analyze subsequent effects on male aggression and courtship. Expression of the murine DNA methyltransferase 3a (DNMT3a) in *Drosophila* has previously been demonstrated to methylate cytosine residues at levels three fold greater than endogenous concentrations^65–67^. In addition to methylating cytosine residues, DNMT3a increases histone H3K9 methylation and reduces histone H3S10 phosphorylation^67^. As H3K9me is associated with transcriptionally inactive heterochromatin formation^68, 69^ and H3S10 phosphorylation indicates transcriptionally active loci^70^, alterations at these residues by DNMT3a expression could also generate the formation of heterochromatin leading to a repression of transcriptional activity in OA neurons.

**Figure 5:**
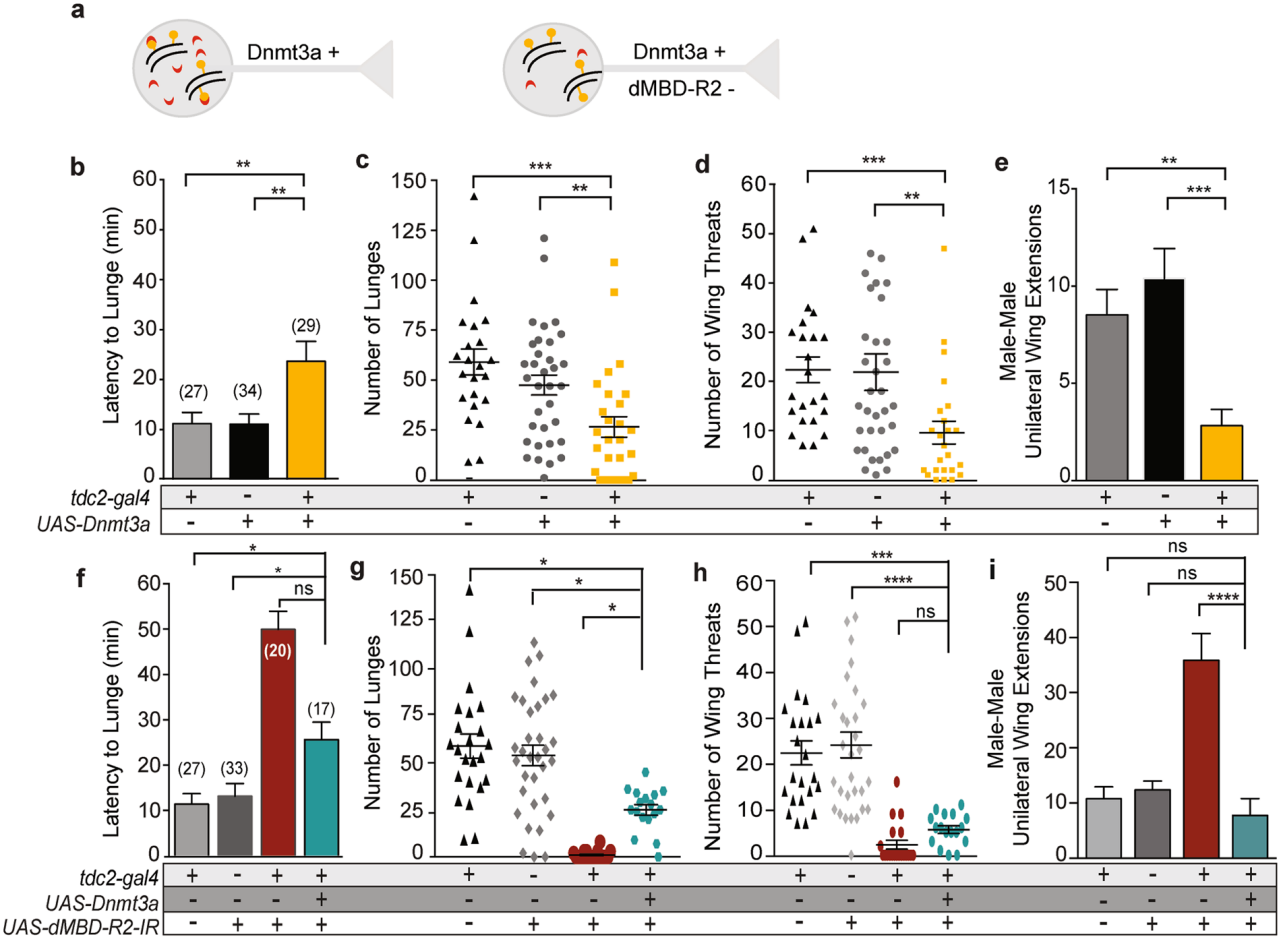
Hypermethylation of the OA neuronal genome decreases aggression and male-male courtship. (**a**) A neuron with increased levels of methylated DNA (orange) and normal levels of dMBDR2 proteins (red, left) is compared to a neuron with a hypermethylated genome and a concurrent reduction in dMBD-R2 levels (right). (**b**–**e**) Fights between pairs of males with genomic (5mC) hypermethylation in OA neurons through the expression of mouse DNA methyltransferase (Dnmt3a) in OA neurons *(tdc2-gal4;UAS-Dnmt3a)* and transgenic controls, *UAS-Dnmt3a/*+ or *tdc2-gal4/*+. (**b**) The onset of aggression as measured by the latency to lunge was significantly delayed in experimental males as compared to controls; all statistical tests are Kruskal Wallis with Dunn’s multiple comparison test except where noted, ****p < 0.0001, ***p < 0.001, **p < 0.01*p < 0.05). (**c**) Pairs of *tdc2-gal4;UAS-Dnmt3a* males displayed a reduction in lunge number as compared to pairs of control males. (**d**) A reduction in the number of wing-threats in males with hypermethylated DNA is observed as compared to transgenic controls. (**e**) Male-male courtship significantly decreased between experimental (*tdc2-gal4;UAS-Dnmt3a*) and control males. The Rout method with a Q set to 0.2% was used to identify four outliers. (**f**–**i**) Fights between pairs of males with a hypermethylated OA neuron genome and a reduction in dMBD-R2 *(tdc2-gal4;UAS-Dnmt3a/UAS-dMBD-R2-RNAi*). males with reduced dMBD-R2 levels and transgenic controls. (**f**) Latency to lunge did not significantly decrease with a reduction of dMBD-R2 (^ns^ *P* = 0.1418). (**g**) Lunge number significantly increased with a reduction of dMBD-R2. **(h)** Number of wing threats were unchanged in *tdc2* < *Dmnt3a/* < *dMBD-R2-RNAi* males (^ns^ *P* = > 0.9999). **(i)** The number of male-male wing extensions significantly decreased with a reduction of dMBD-R2. Error bars indicate SEM.

We used the same *UAS-Dnmt3a* transgene to drive DNMT3a expression in OA neurons (*tdc2;Dnmt3a)* and asked first if hypermethylation of this neuronal genome (Fig. 5a) would alter male behavior. We predicted if the decrease in male aggression and increase in male-male courtship exhibited by *tdc2:dMBD-R2-RNAi* males occurs via methylation-independent effects, adding new methylation targets would not alter the behavioral phenotypes.

Our results suggest dMBD-R2 plays a role in interpreting both methylation-dependent and methylation independent gene expression. Experimental *tdc2;Dnmt3a* males displayed a significant increase in the latency to initiate aggression (Fig. 5b) and a reduction in lunge numbers and wing threats (Fig. 5c,d) as compared to controls suggesting increased methylation of the OA neuronal genome does alter the MBD-R2 regulation of chromosomal regions that promote aggression. Strikingly, the expression of Dnmt3a in OA neurons significantly decreased the number of male-male courtship bouts (Fig. 5e). This result contrasts with the increase in male-male courtship observed by a reduction of dMBD-R2 (Fig. 3h) and suggests a methylation-dependent component. With this information, we examined if reducing dMBD-R2 levels in conjunction with Dnmt3a expression alters the hyper-methylation-mediated decrease in aggression by generating *tdc2;Dnmt3a/dMBD-R2-RNAi* males (Fig. 5f–i). Two aggression parameters, latency to lunge and number of wing threats did not significantly differ when dMBD-R2 levels were decreased in the hyper-methylated OA neuron genome (Fig. 5f,h), however lunge number and number of male-male courtship events did significantly increase and decrease respectively (Fig. 5g,i). These results suggest interactions between dMBD-R2 and Dnmt3a-induced hypermethylation states in determining overall aggressive behavioral output.

## Discussion

Natural selection of heritable genetic variation provides a key mechanism for evolutionary adaption to changes in the environment. An organism’s genetic composition constrains its physiological, morphological and behavioral plasticity thereby influencing fitness landscapes across environmental gradients. However, recent years have witnessed a growing body of literature documenting an epigenetic framework that deconstrains these limits on plasticity. Epigenetic processes such as methylation and histone modifications regulate gene expression by structuring and remodeling of chromatin states in response to changes in the environment, allowing dynamic shifts in organismal physiology and behavior. For example, changes in biotic or abiotic stress^71–75^ as well as social and behavioral experiences^76–78^ can induce targeted restructuring of genomic methylation states. While it is thought that the primary importance of epigenetic marks in evolution is to ensure developmental plasticity, a role for methylation modifications in combination with genetic variation may facilitate genetic divergence and ultimately contribute to reproductive isolation and speciation^10, 79, 80^.

Although methylation levels are low and sparsely distributed in *Drosophila*, both ^5^CpH methylation (H = A/C/T/G), as well as N^6^A methylation states are associated with the regulation of transcriptional activity^21, 24, 81^. Furthermore, the methyl-CpG binding domain (MBD) retains considerable functional identity across species^39^. As MBD protein family members translate these epigenetic marks to appropriate functional states, we simply asked if MBD proteins in *Drosophila* contribute to setting the behavioral strategies associated with reproductive isolation as well as survival behaviors. We quantified courtship displays including unilateral wing extensions (singing), attempted copulations (abdomen bending), and copulation success of Canton S wildtype males, transgenic controls, and males with a reduction in endogenous MBD proteins. All males displayed singing and attempted copulation behaviors to both conspecific and inter-specific females supporting previous reports that *D. melanogaster* males are at least partially indiscriminate in courting females^82–85^. Unlike controls however, males with a reduction in MBD protein levels, court inter-specific females at levels higher in the case of *D*. *virilis* or the same with *D*. *yakuba* as compared to conspecific females suggesting an additional filter on species discrimination has been removed. Does this increase in inter-specific courtship have any bearing on copulation success, the ultimate determinant of sexual selection? In the assay with *D*. *virilis* and conspecific females, the answer is yes, as mating success of dMBD-R2 deficient males solely in OA neurons is significantly reduced.

What transcriptional activity or genomic landscape might be altered in OA neurons as a result of a reduction in MBD proteins? We recently demonstrated that OA neurons act as second-order transducers in Gr32a-mediated chemosensory-information pathway^16^. The reduction in the number of successful mating events with conspecific females as well as the increase in male-male courtship within the aggression paradigm by MBD-deficient males may reflect an impaired ability to reliably process and respond to chemosensory identification cues^16, 86^. Together, our results suggest a model in which dMBD-R2 plays a key role in interpreting gene activity involved in processing species- and sex-specific chemosensory cues within the OA neuron genomic landscape.

In summary, as epigenetic modifications serve as an interface between multilayered gene-environment interactions, dynamic epigenetic restructuring in response to changes in external conditions (including social interactions) may induce transient or long-term changes in cellular function and/or organismal behavior. Our results highlight the critical role MBD proteins play in mediating organismal fitness in the context of survival and reproductive behaviors; and pave the way to address the mechanics of epigenomic shifts that may mediate reproductive isolation through phenotypic plasticity in chemosensory processing and mating behavioral strategies.

## Electronic supplementary material

Supplementary Figure Legends and Figures
