# Supplementary material for: Methyl-CpG binding domain proteins inhibit interspecies courtship and promote aggression in Drosophila
Source: Sci Rep. 2017 Jul 14;7:5420. doi: 10.1038/s41598-017-05844-6 (PMC5511146; doi:10.1038/s41598-017-05844-6)

SREP-16-47529

Methyl-CpG binding domain proteins inhibit interspecies courtship and promote aggression in *Drosophila*

Tarun Gupta[1†], Hannah R. Morgan[2†], Jonathan C. Andrews[2], Edmond R. Brewer[2], and Sarah J. Certel[1,2*]

Supplemental Figure 1: *D. melanogaster* males preferentially court conspecific females

(a-d) Courtship patterns displayed by Canton S males toward a conspecific (CS) and interspecific *(D. virilis;* labeled DV) female were quantified. Mann-Whitney test was used unless otherwise specified, ****p<0.0001, ***p<0.001, *p<0.05. n=16 **(a)** Canton S males spent the majority of the time courting the conspecific female. **(b)** The number of unilateral wing extensions (singing) toward conspecific females was higher than towards inter-species females. **(c)** The duration of wing extensions to DV and CS females did not differ in transgenic control males (^{ns}P = 0.8512; Kruskal-Wallis Test with Dunn's multiple comparison). The average bout length of unilateral wing extensions towards CS females was longer than to DV females. (**d**) Canton S males did not exhibit a preference in the average number and fractional distribution of copulation attempts to DV and CS females. Unpaired two-tailed t test with Welch's correction: t=1.321, df=22.36, P value: 0.1966. **(e)** The latency to copulate with the CS female and the percentage of assays that resulted in a successful conspecific mating event. **(f-k)** The courtship patterns displayed by *CS; Trh-Gal4/+* males toward a conspecific (CS) and a *D. virilis* female were quantified. n=16 **(f)** *CS; Trh-Gal4/+* males spent more time courting the conspecific female. (P< 0.05). **(g)** The number of unilateral wing extensions (singing) toward conspecific females and interspecies females did not differ, (^{ns}P=0.06). **(h)** The duration of wing extensions to DV and CS females did not differ in *CS; Trh-Gal4/+* males (^{ns}P = 0.1031). **(i)** *CS; Trh-Gal4/+* males did not exhibit a preference in the average number and fractional distribution of copulation attempts to DV and CS females (^{ns}P = 0.9183). (**k**) The latency to copulate with the CS female and the percentage of assays that resulted in a successful conspecific mating event. Data is represented as mean $\pm$ S.E.M.

Supplemental Figure 2: Males with reduced MBD-2/3 levels in OA neurons court *D. virilis* females and exhibit reduced conspecific mating

(a) dMBD-2/3 targeted knockdown in OA neurons through the *UAS-Gal4* system. **(b-e)** Quantification of courtship behavioral patterns displayed by males with a reduction in dMBD-

2/3 *(tdc2-Gal4;UAS-dMBD-2/3-RNAi)* and transgenic control males towards conspecific (CS) and interspecific *(D. virilis;* labeled DV) females. Mann-Whitney test was used unless otherwise specified, ****p<0.0001, ***p<0.001, **p<0.01, *p<0.05. n = 18 (*tdc2-Gal4/+*), 12 (*UAS-MBD-2/3-IR/+*), and 26 (*tdc2-Gal4;UAS-MBD-2/3-IR*). **(b)** Males with reduced levels of dMBD-2/3 in OA neurons spent the majority of the time (74%) courting DV females as compared to transgenic controls. (**c**) The average number and the proportion of abdomen bends toward DV females were not significantly higher in dMBD-2/3 deficient males than copulation attempts to conspecific females. **(d)** The number of unilateral wing extensions toward interspecific females was higher than towards conspecific females in dMBD-2/3-reduced males as compared to control males. **(e)** Courtship index (C.I.) calculated as total time spent courting either female as a fraction of total scoring period was significantly lower between experimental and control males. **(f)** The percent of assays that resulted in a successful conspecific mating event was decreased significantly in *tdc2-Gal4;UAS-MBD-2/3-IR* males as compared to transgenic controls (N-1 Chi-square test for proportions). Error bars indicate SEM.

Supplemental Figure 3: Reducing MBD-R2 through a second RNAi interference line decreases male aggression

(a–d) Fights between males with reduced dMBD-R2 levels using a second RNAi line (BL 27029) in OA neurons *(tdc2-Gal4;UAS-dMBD-R2-RNAi^{27029})* and individual transgenic controls, *UAS-MBD-R2-IR^{27029}/+* or *tdc2-Gal4/+*. **(a)** Latency to first lunge was significantly increased in *tdc2;dMBD-R2-RNAi^{27029}* males as compared to controls (****p<0.0001, ***p<0.001, **p<0.001, *p<0.05; Kruskal Wallis with Dunn's multiple comparison test except where noted). The n value for each genotype is 26, 15, and 13. **(b)** Males with reduced dMBD-R2^{27029} levels in OA neurons lunged significantly less than controls. **(c)** dMBD-R2^{27029}-deficient males display reduced wing threats as compared to transgenic controls. **(d)** Male-male courtship wing extensions decreased in pairs of dMBD-R2^{27029} deficient males than controls which may suggest the reduction of dMBD-R2 levels by the dMBD-R2^{27029} line is effective enough to decrease aggression but not prevent the inhibition of male-male courtship. **(e)** The ratio of male courtship events per lunge number was significantly decreased in dMBD-R2^{27029} deficient males as compared to transgenic controls. Error bars indicate SEM.

Supplemental Figure 4: Tdc2 expression decreases within specific neurons in dMBD-R2 deficient males

(a) *UAS-dMBD-R2-IR/+* male brain labeled with anti-Tdc2, arrow and arrowhead are cells quantified for Tdc2 fluorescence. **(b)** tdc2>dMBD-R2-RNAi male brain labeled with anti-Tdc2. **(c)** Quantification of fluorescence indicates the OA neuron located in the subesophageal ganglion zone (arrow) exhibits less fluorescence than the control neuron (unpaired t-test, P=0.023). The fluorescence between control and MBD-R2-deficient brains of the ventrolateral OA neuron (arrowhead) is not significantly different (unpaired t-test, P=0.497). Error bars indicate SEM.

Supplemental Figure 1: Courtship rates toward conspecific and interspecific females in wildtype and transgenic control males

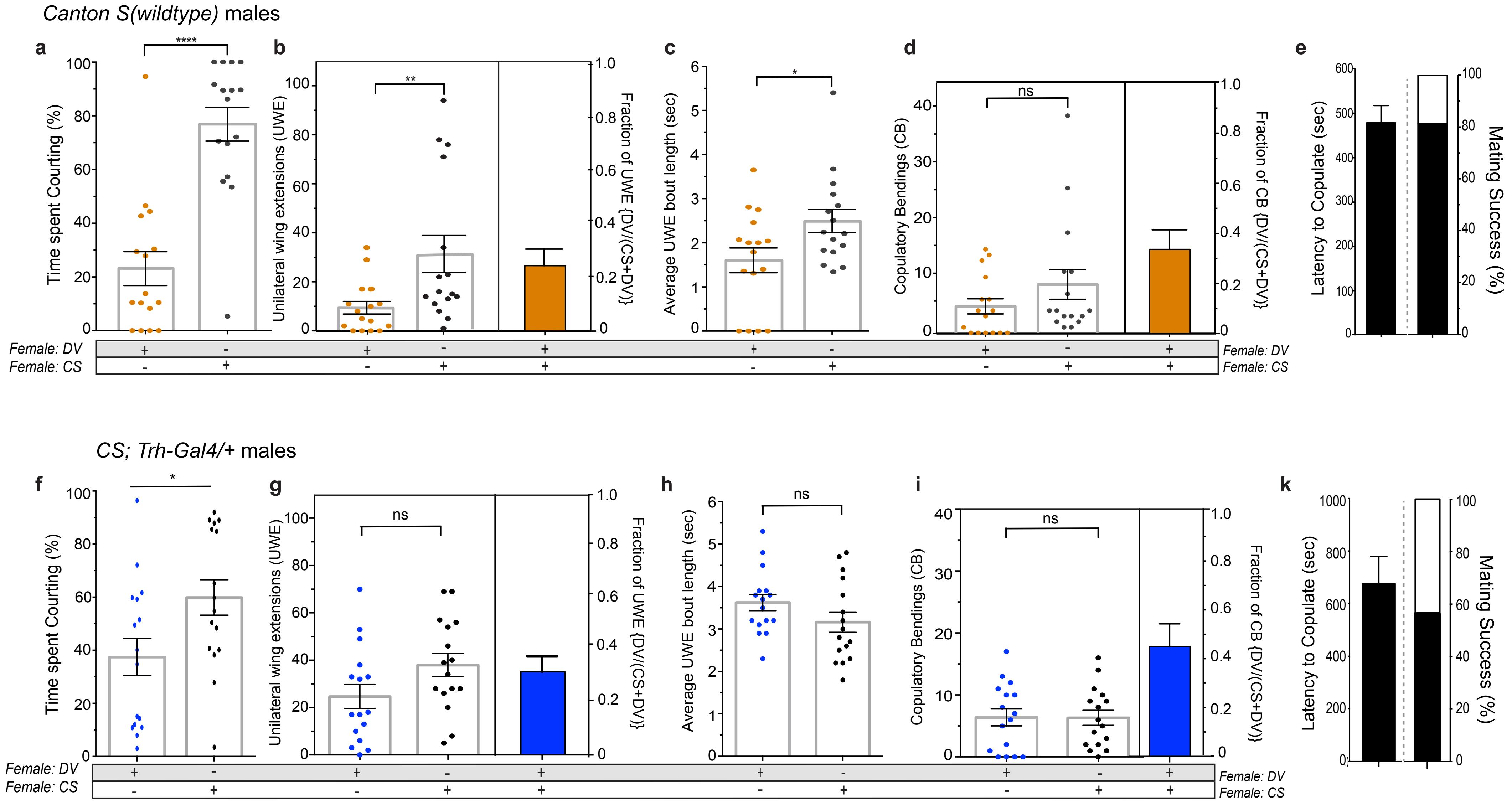

SFigure 2: dMBD-2/3-deficient males display court D. virilis females and exhibit reduced conspecific mating

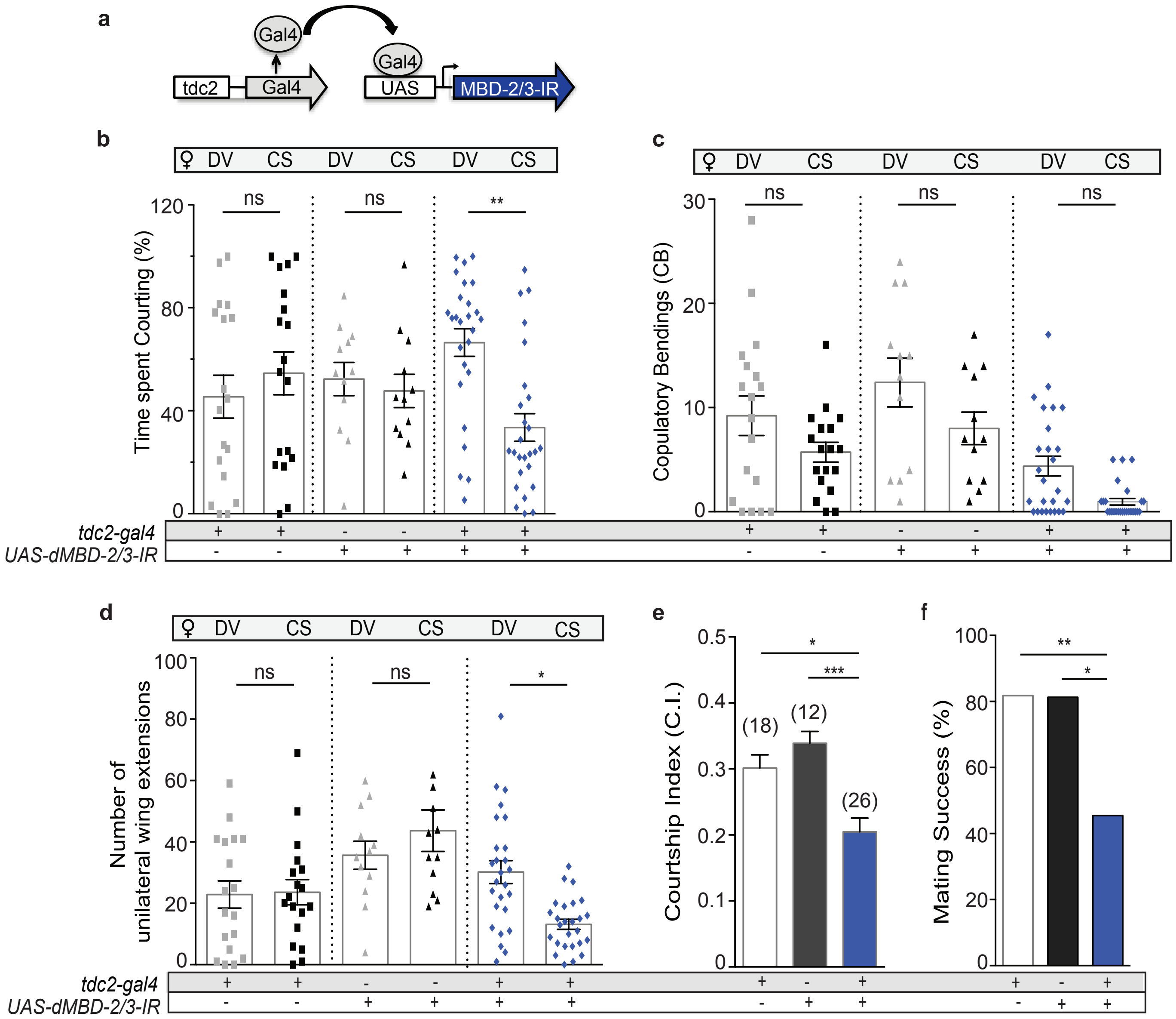

Supplemental Fig. 3: Reducing MBD-R2 levels with a second RNAi line decreases male aggression

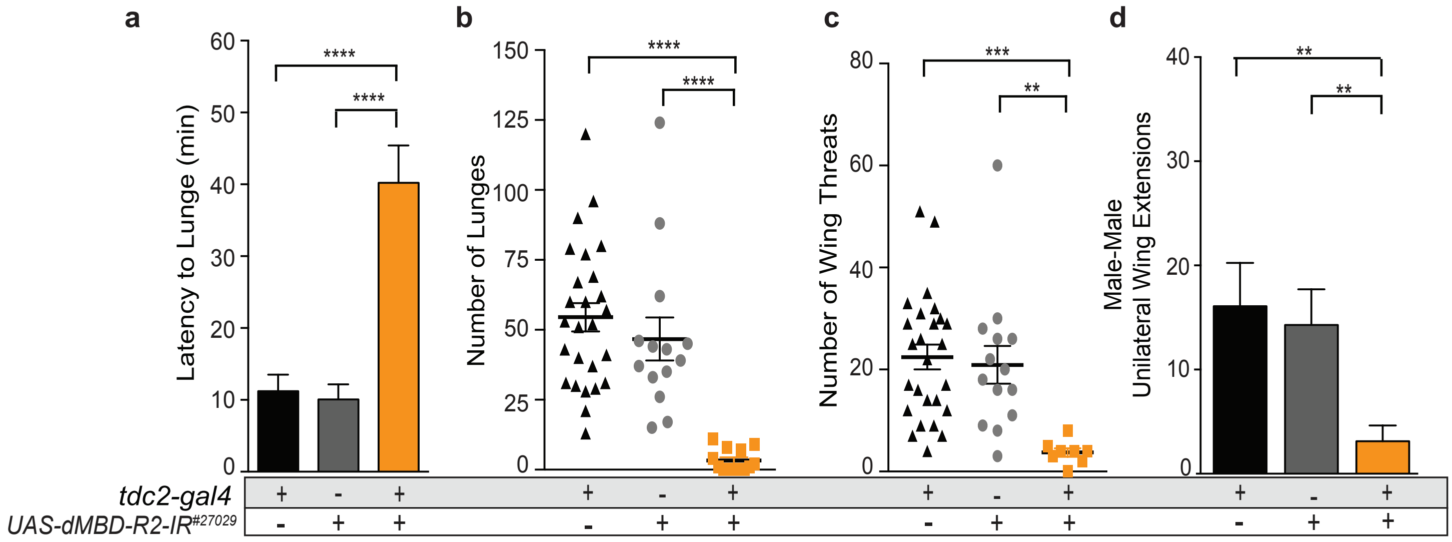

Supplemental Figure 4: Tdc2 expression decreases within specific neurons in dMBD-R2 deficient males

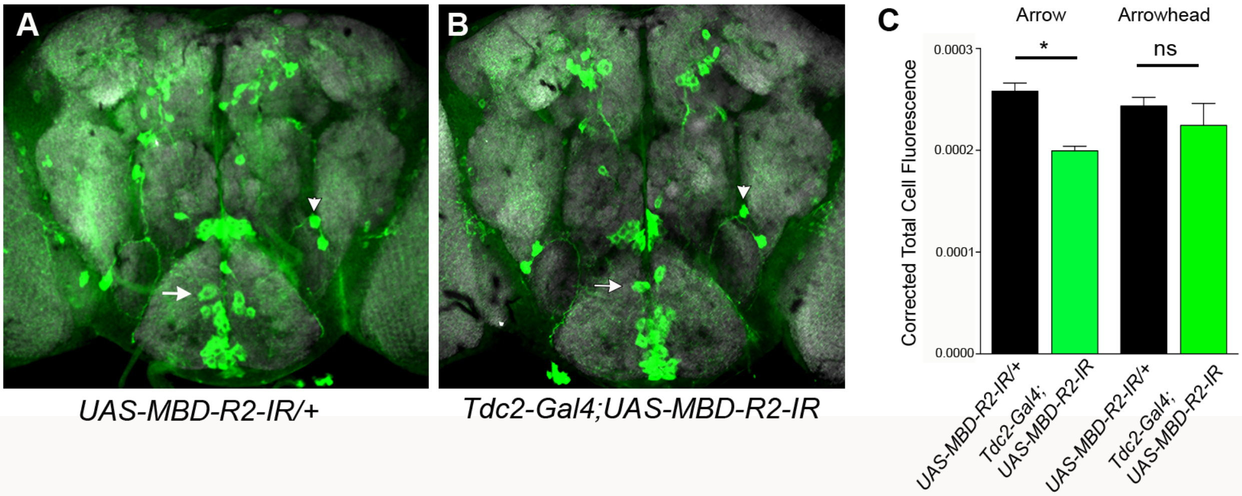

Supplement: Supplementary file 1 — Supplementary Figure Legends and Figures [file 41598_2017_5844_MOESM1_ESM.pdf]
